# Hypofractionated radiotherapy for refractory or relapsed aggressive B-cell lymphoma in the rituximab era

**DOI:** 10.1186/s12885-024-11837-2

**Published:** 2024-01-13

**Authors:** Cheng Huang, Tian-Lan Tang, Yan-Yan Qiu, Yu-Ping Lin, Si-Lin Chen, Rui-Zhi Zhao, Gui-Qing Shi, Si-Qin Liao, Jin-Hua Chen, Hai-Ying Fu, Jian-Zhi Liu, Ben-Hua Xu, Ting-Bo Liu, Yong Yang

**Affiliations:** 1 Department of Radiation Oncology, Fujian Key Laboratory of Intelligent Imaging and Precision Radiotherapy for Tumors (Fujian Medical University), Clinical Research Center for Radiology and Radiotherapy of Fujian Province (Digestive, Hematological and Breast Malignancies ), Fujian Medical University Union Hospital, Fuzhou, 350001 P. R. China; 2 Department of Hematology, Fujian Provincial Key Laboratory On Hematology, Fujian Medical University Union Hospital, Fujian Institute of Hematology, Fuzhou, P. R. China; 3 Department of PET/CT, Fujian Medical University Union Hospital, Fuzhou, P. R. China; 4 Follow-Up Center, Fujian Medical University Union Hospital, Fuzhou, P. R. China; 5 Department of Hematology, The Third Affiliated People’s Hospital of Fujian University of Traditional Chinese Medicine, The Third People’s Hospital of Fujian Province, Fuzhou, P. R. China; 6 Department of Otorhinolaryngology, Fujian Medical University Union Hospital, Fuzhou, P. R. China

**Keywords:** Radiotherapy, Hypofractionated, Refractory, Relapsed, B-cell lymphoma

## Abstract

**Background:**

Radiotherapy (RT) is an effective and available local treatment for patients with refractory or relapsed (R/R) aggressive B-cell lymphomas. However, the value of hypofractionated RT in this setting has not been confirmed.

**Methods:**

We retrospectively analyzed patients with R/R aggressive B-cell lymphoma who received hypofractionated RT between January 2020 and August 2022 at a single institution. The objective response rate (ORR), overall survival (OS), progression-free survival (PFS) and acute side effects were analyzed.

**Results:**

A total of 30 patients were included. The median dose for residual disease was 36 Gy, at a dose per fraction of 2.3–5 Gy. After RT, the ORR and complete response (CR) rates were 90% and 80%, respectively. With a median follow-up of 10 months (range, 2–27 months), 10 patients (33.3%) experienced disease progression and three died. The 1-year OS and PFS rates for all patients were 81.8% and 66.3%, respectively. The majority (8/10) of post-RT progressions involved out-of-field relapses. Patients with relapsed diseases, no response to systemic therapy, multiple lesions at the time of RT, and no response to RT were associated with out-of-field relapses. PFS was associated with response to RT (*P* = 0.001) and numbers of residual sites (*P* < 0.001). No serious non-hematological adverse effects (≥ grade 3) associated with RT were reported.

**Conclusion:**

These data suggest that hypofractionated RT was effective and tolerable for patients with R/R aggressive B-cell lymphoma, especially for those that exhibited localized residual disease.

## Background

Worldwide, aggressive B-cell lymphoma is the most common subtype of non-Hodgkin lymphoma (NHL) [1, 2]. The standard first-line R-CHOP (rituximab, cyclophosphamide, doxorubicin, vincristine, and prednisone) immunochemotherapy achieves long-term remission in approximately two-thirds of adult patients and others suffer from primary refractory or relapsed (R/R) lymphoma after an initial response [1, 3]. Although many efforts have been made to improve patient survival over the past two decades, including increase dose-send/intensity of systemic therapy, maintenance therapy, and R-CHOP plus a novel drug (R-CHOP + X), the standard of care for unspecified patients has not changed [4, 5]. Hence, many new therapeutic approaches have been developed that focus on R/R diseases [6–10].

The standard of care for patients with late relapse (> 12 months) is high-dose chemoimmunotherapy with autologous stem-cell transplantation (ASCT) if the disease is responsive to salvage regimens [1, 5, 7, 11, 12]. However, because of aging, concurrent morbidities, and chemoresistance, only 25% patients are considered candidates for transplantation [7, 13–16]. Autologous chimeric antigen receptor (CAR) T-cell therapy, a gene-modified cellular treatment, represents a major paradigm shift in the management of R/R B-cell lymphomas [6, 17, 18]. To avoid a delay in constitutes infusion, several retrospective trials have used radiotherapy (RT) as a bridging or salvage strategy for CAR T-cell therapy, with reported response rates of 80–88% [19–26].

The efficacy of RT to improve local control of aggressive B-cell lymphoma is well established [27–34]. In addition, several large database analyses have shown improved survival with the addition of RT after controlling for confounding factors through multivariate analysis in the rituximab era [35–38]. Recently, in a comprehensive retrospective study (British Columbia Cancer Lymphoid Cancer Database), the positron emission tomography (PET)-positive sites of some patients who received RT for nonprogressive disease showed results comparable to those with PET-negative findings [39]. Additionally, the predominant pattern of relapse following systemic therapy (including first-line chemotherapy, ASCT, and CART) often involve sites of initial [21, 40–43]. These predictable patterns of relapse emphasize the utility of RT to improve local control to all sites of disease. However, an interval of over 4 weeks induced by RT, which can delay systemic salvage therapies for R/R patients, is a crucial concern for hematologists.

Regardless of a consolidation or salvage setting, conventional RT has been shown to be a safe and promising tool to help control the disease; however, the clinical value of hypofractionated RT is still poorly understood. The aim of this study was to investigate the outcomes and toxicity of hypofractionated RT in R/R patients in a single facility.

## Methods

### Eligibility and study population

Patients with R/R aggressive B-cell lymphoma between January 2020 and August 2022 at a single institution were retrospectively reviewed (*n* = 59). The eligibility criteria included R/R patients who had received hypofractionated RT prior to or after salvage systemic treatment. Patients who had received conventional fractionated RT (*n* = 17), showed central nervous system (CNS) involvement, or had primary CNS lymphoma (*n* = 12) were excluded. Eventually, 30 patients were eligible for the final analysis.

### Evaluation and definition

Patients were initially staged according to the Ann–Arbor staging system and scored using the international prognostic index. The tumor response was evaluated after completion of chemotherapy, RT, or a combination of chemotherapy and RT. Complete response (CR) was defined as the elimination of all signs of disease in the clinical and imaging examinations. Refractory disease was defined as an incomplete response after primary chemotherapy. Relapsed disease was defined as new disease found on imaging or biopsy after CR. All patients were re-evaluated with CT scan before RT, and 26 patients (86.7%) also underwent a PET scan. Adverse events were evaluated using CTCAE (common terminology criteria for adverse events) version 5.0.

In- and out-of-field relapses for RT were defined based on imaging or biopsy. If the failure occurred in the same area of the lymph node that had been irradiated, it was deemed to be an in-field relapse. If the failure occurred in an area of the distant lymph node other than outside the irradiated area, it was considered an out-of-field relapse. Out-of-field relapse after RT was categorized as pre-existing sites only, new sites only, or both. Relapse at pre-existing sites was defined as a recurrent disease at the same sites before first-line chemotherapy. Relapse at new sites was identified as a recurring disease outside of sites prior to first-line treatment.

### Treatment

Immunochemotherapy was considered the primary treatment of aggressive B-cell lymphoma. All patients were treated with immunochemotherapy and the regimens were R-CHOP (*n* = 26) and dose-adjusted EPOCH-R (etoposide, prednisone, vincristine, cyclophosphamide, doxorubicin, rituximab, *n* = 4). The median number of chemotherapy cycles was 4 (range: 3–8).

Radiotherapy was given with a 6-MV linear accelerator. As directed by the International Lymphoma Radiation Oncology Group (ILROG), involved-site radiation therapy (ISRT) was administered [44, 45]. PET or magnetic resonance imaging (MRI) were obtained and co-registered with planning CT to improve delimitation of the treatment volume. Gross tumor volume (GTV) was defined as residual diseases in PET/CT or CT. Adjacent nodal diseases that responded to chemotherapy may be included in the clinical target volume (CTV), as long as their inclusion was not associated with significant toxicity. A 3–7-mm margin was added to the GTV and CTV to generate the corresponding planning gross target volume (PGTV) and planning target volume (PTV), respectively. The median dose for GTV was 36 Gy (range: 30–39 Gy), at a dose per fraction of 2.3–5 Gy. Since December 2021, 24 Gy to PTV with a simultaneous integrated boost 36 Gy to PGTV in 12 fractions were widely applied at our institution (*n* = 23, 76.7%). The numbers of treated sites was defined as the numbers of radiation field required to treat all target volumes. Organs at risk (OAR) included the parotid glands, larynx, spinal cord, lungs, heart, kidney, liver, small intestine, bladder, rectum, and head of the femur.

### Statistical analysis

Continuous variables were reported in medians and ranges, and categorical variables were reported in frequencies and percentages. The primary endpoint was response to RT, defined as either CR or partial response (PR); secondary endpoints included progression-free survival (PFS) and overall survival (OS). PFS was defined as the period from the date of RT to the date of any relapse, progression, last follow-up, or death from any cause. OS was calculated from the date of RT to the date of death from any cause or until the last follow-up. PFS and OS were estimated using the Kaplan–Meier method and compared using log-rank tests stratified by prognostic factors. *P* < 0.05 was considered to indicate statistically significant differences. All statistical analyses were performed using SPSS (version 26.0; IBM Corporation, Armonk, NY, USA) and R (version 3.5.3) software.

## Results

### Clinical characteristics

Final analyses were conducted on 30 patients, and the baseline clinical features and initial treatments are summarized in Table 1. The median age was 55 years (range: 19–79 years) and 60% patients were female. At initial diagnosis, extranodal involvement was present in 76.7% patients, bulky disease (≥ 7.5 cm) was present in 46.7%, and the majority had advanced-stage disease (stage III/IV, 63.3%). The distribution of medical histology is as follows: diffuse large B-cell lymphoma not otherwise specified (DLBCL-NOS, *n* = 20); primary mediastinal large B-cell lymphoma (PMBL, *n* = 6); transformed DLBCL (*n* = 2); primary breast DLBCL (*n* = 1); and high-grade B-cell lymphoma (MYC, BCL2, and BCL6 rearrangement, *n* = 1).

**Table 1:** Patient characteristics and treatment at initial presentation

Characteristics	Patients	
	Number	Percent
Age, median (range)	55.5 (19–79)	
Sex		
Female	18	60%
Male	12	40%
Ann Arbor Stage		
I/II	11	36.7%
III/IV	19	63.3%
Extranodal involvement		
Yes	23	76.7%
No	7	23.3%
Bulky disease, cm		
≥ 7.5	14	46.7%
< 7.5	16	53.3%
Histology		
DLBCL-NOS	20	66.7%
Primary mediastinal B-cell lymphoma	6	20%
Transformed DLBCL	2	6.7%
High-grade B-cell lymphoma	1	3.3%
Primary breast B-cell lymphoma	1	3.3%
Initial systemic regimen		
R-CHOP	26	86.7%
DA-EPOCH-R	4	13.3%

*Abbreviations*: *DLBCL-NOS* diffuse large B-cell lymphoma not otherwise specified, *R-CHOP* rituximab, cyclophosphamide, doxorubicin, vincristine, and prednisone, *DA-EPOCH-R* dose-adjusted etoposide, prednisone, vincristine, cyclophosphamide, doxorubicin, rituximab, *RT* radiotherapy

### Radiotherapy outcomes

Baseline patient characteristics at the time of RT are listed in Table 2. Prior to RT, most patients experienced PR after initial therapy (86.7%), and the remaining 4 (13.3%) patients had progressive disease (PD) after chemotherapy. Second-line chemotherapy was used in 7 (23.3%) patients, and 1 (3.3%) patient received third-line treatment before RT. Three-quarters of RT patients exhibited localized disease (76.7%), with a total of 45 treated sites. The median maximum diameter of residual lesions was 4.5 cm, and the median volumes of GTV and CTV were 53 mL and 372 mL, respectively.

**Table 2:** RT characteristics and treatment response (*n* = 30)

Characteristics	Patients	
	Number	Percent
Refractory/relapsed		
Refractory	27	90%
Relapsed	3	10%
Numbers of residual sites		
1	23	76.7%
≥ 2	7	23.3%
Extranodal involvement		
Yes	18	60%
No	12	40%
Maximum diameter of residual tumor, median (range)	4.5 cm (1–9 cm)	
Lines of chemotherapy before RT		
1	22	73.3%
2	7	23.3%
3	1	3.3%
RT dose and fractionation		
36 Gy/12f	23	76.7%
30 Gy/6f	3	10%
39.1 Gy/17f	3	10%
30 Gy/10f	1	3.3%
Numbers of treated sites	45	100%
1	22	73.3%
2	5	16.7%
3	1	3.3%
4	1	3.3%
6	1	3.3%
RT modality		
IMRT	9	30%
VMAT	21	70%
Response to RT		
CR	24	80%
PR	3	10%
PD	3	10%

*Abbreviations*: *RT* radiotherapy, *IMRT* intensity-modulated radiation therapy, *VMAT* volumetric-modulated arc therapy, *CR* complete response, *PR* partial response, *PD* progressive disease

**Fig. 1:**
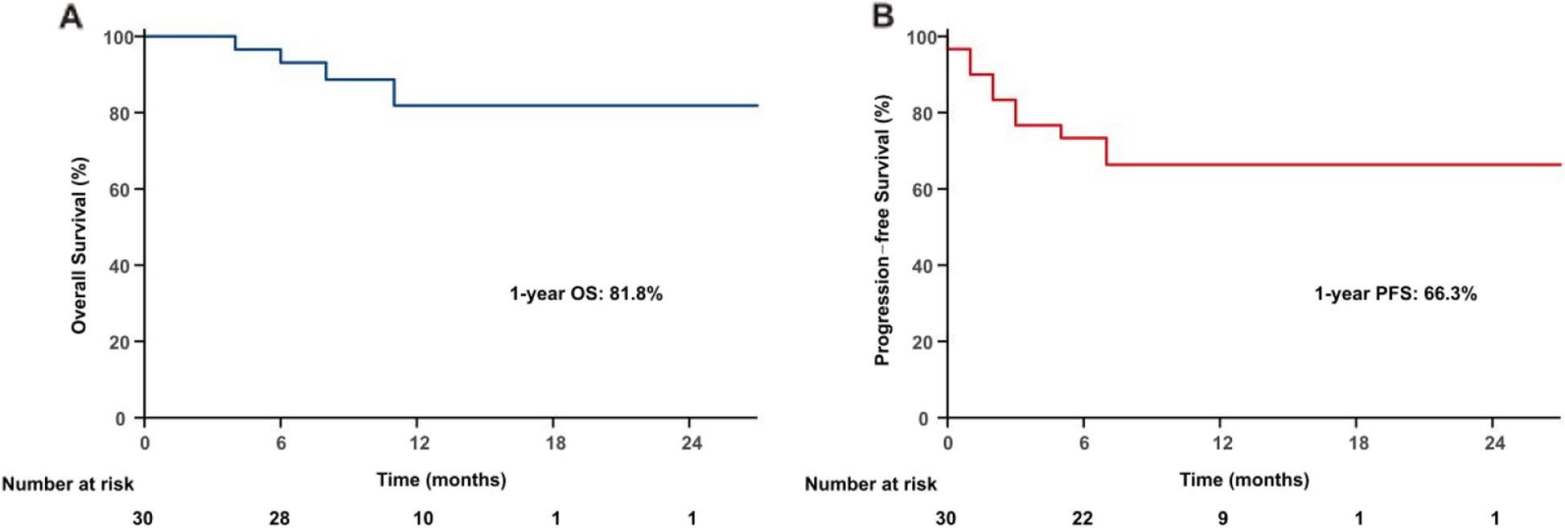
OS (A) and PFS (B) for all patients

**Fig. 2:**
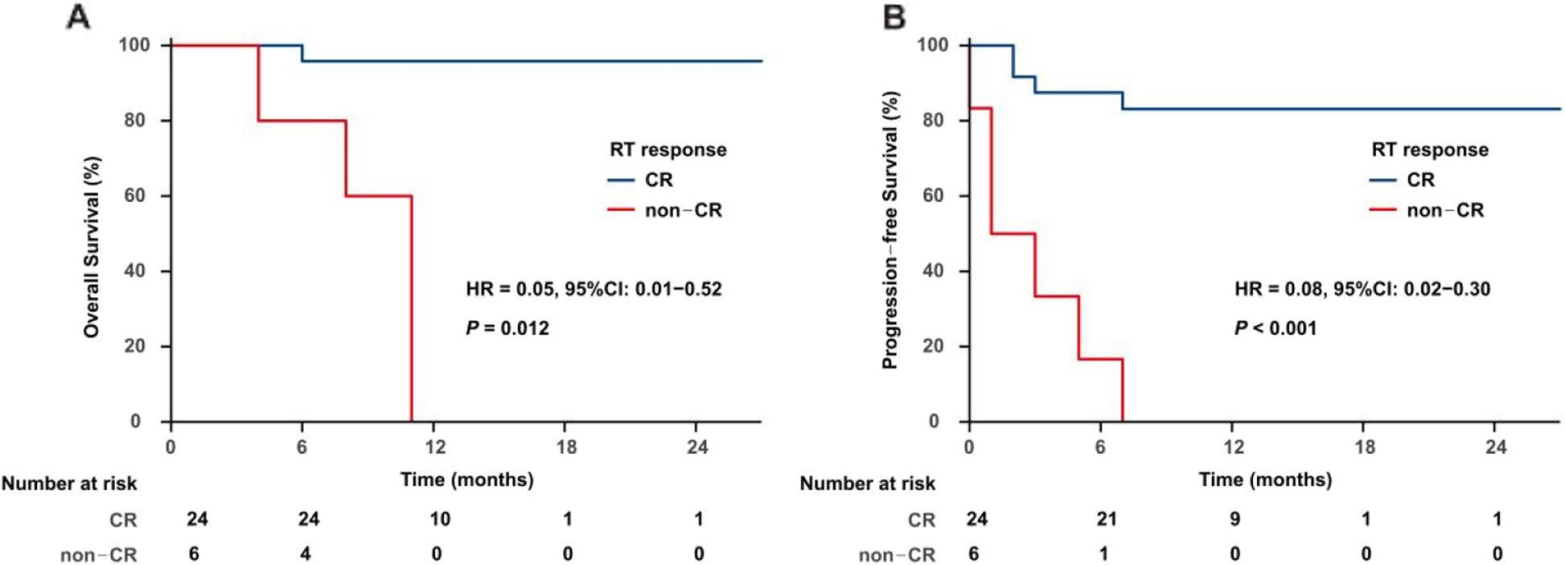
OS and PFS stratified by RT response. OS (**A**) and PFS (**B**) were worse when patients achieved non-CR after RT

**Fig. 3:**
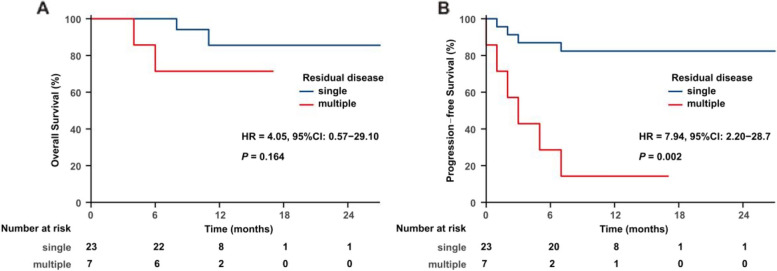
OS and PFS stratified by number of residual diseases at the time of RT. OS (**A**) and PFS (**B**) were worse when patients had multiple residual disease

All patients received either intensity-modulated radiation therapy (IMRT) or volumetric-modulated arc therapy (VMAT). Subsequently, 19 patients received salvage chemotherapy. Among the 30 evaluable patients, 27 (90%) achieved an objective response after the completion of RT: 24 (80%) CR and 3 (10%) PR. In the 45 lesions being treated, 39 (86.7%) achieved CR, 4 (8.9%) had PR, and 2 (4.4%) exhibited PD. Specifically, among the 8 patients who had multiple lesions at the time of RT, the CR rate was 87% (20/23) for a total of 23 treated sites. With a median follow-up of 10 months (range, 2–27), 10 of the 30 (33.3%) patients experienced disease progression, and three patients died. The 1-year OS and PFS rates for all patients were 81.8% and 66.3%, respectively (Fig. 1). The corresponding 1-year OS and PFS rates for patients who obtained CR after RT were 95.8% and 83.1%, respectively, and 0% (*P* = 0.001, Fig. 2A) and 0% (*P* = 0.001, Fig. 2B) for patients who had not. The 1-year PFS rate was 82.4% for patients who had a single lesion at the time of RT compared with a 1-year PFS rate of 14.3% for patients who had multiple lesions (*P* < 0.001); there was no statistically significant difference in OS (*P* = 0.132) (Fig. 3).

### Failure patterns and associated factors

**Fig. 4:**
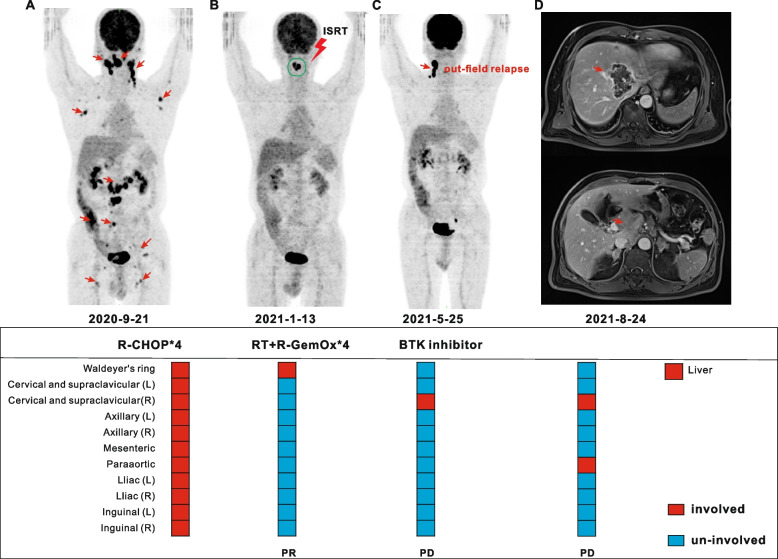
A 71-year-old male patient experienced out-of-field relapse after RT. He was diagnosed with DLBCL (stage III), and the initial involved sites included Waldeyer's ring, bilateral cervical, axillary, mesenteric, paraaortic, bilateral iliac, and inguinal sites (**A**). Patient achieved PR (residual lesions in Waldeyer's ring) after four cycles of R-CHOP, and received RT and four cycles of R-GemOx (rituximab, gemcitabine, and oxaliplatin) (**B**). After RT and second-line chemotherapy, patients experienced out-of-field relapse in the right cervical (**C**). Then, he received Bruton’s tyrosine kinase (BTK) inhibitor, but still experienced disease progression in the liver and paraaortic region (**D**)

For the entire cohort, failure analysis showed that the majority of post-RT progressions involved out-of-field relapses (Table 3). After RT, 2 (6.7%) relapses were completely in-field, 3 (10%) were a combination of in- and out-of-field relapses, and 5 (16.6%) were completely out-of-field relapses (Fig. 4). All out-of-field relapse patients (*n* = 8) had extranodal involvement; 7 patients had initial stage III/IV disease; and in 5 patients with only out-of-field relapse, all occurred at new sites only after RT. According to univariate analysis, four factors have a significant impact on the incidence of out-of-field relapses: refractory/relapsed (refractory [18.5%] vs. relapsed [100%], *P* = 0.002); response to systemic therapy before RT (yes [19.2%] vs. no [75%]. *P* = 0.019); number of residual sites (single lesion [8.7%] vs. multiple lesions [85.7%], *P* < 0.001); and response to RT (CR [16.7%] vs. no-CR [66.7%], *P* = 0.013).

**Table 3:** Pattern of failure analysis after RT

Characteristics	Patients	
	Number	Percent
Progression		
No	20	66.7%
Yes	10	33.3%
Site of progression		
Pre-existing sites only	2	6.7%
New sites only	4	13.3%
Both	4	13.3%
Site of progression in relation to RT field		
In-field only	2	6.7%
Out-of-field only	5	16.6%
Both	3	10%

*Abbreviations*: *RT* radiotherapy

### RT toxicity and dose to normal tissues

No serious non-hematological adverse effects (≥ grade 3) associated with RT were reported. Radiation-related adverse events included leukocytopenia in three patients (grade 2: two patients, grade 4: one patient) and oral mucositis (grade 2); radiation dermatitis (grade 1); asymptomatic pneumonia (grade 1); and nausea (grade 2) in one patient each, respectively.

Owing to the heterogeneity of RT schemes, we present the DVH statistics for the critical normal tissues of the 23 patients with 36 radiated sites treated with 36 Gy in 12 fractions (Table 4). For five RT sites in the head and neck, the median mean dose (Dmean) to the parotid gland and larynx was 13.2 Gy and 9.7 Gy, respectively, and the median maximal dose (Dmax) to the spinal cord was 14.2 Gy. For 15 RT sites in the thorax (mediastinum and axilla dominate the list), the median lung irradiated by 20 Gy or more (V20) was 4.7%, the median Dmean to the heart was 1.1 Gy, and the median Dmax to the spinal cord was 16.8 Gy. For 10 RT sites in the abdomen, the median V20 of the kidney was 7.47%, and the median Dmax to the small intestine and spinal cord was 33.4 Gy and 15.6 Gy, respectively. For six RT sites in the pelvis, the Dmean to the bladder and rectum was 5.52 Gy and 3.65 Gy, respectively, and the median Dmax to the head of the femur was 16.6 Gy.

**Table 4:** RT characteristics of the 23 patients with 36 sites treated with hypofractionated schemes of 36 Gy in 12 fractions

RT target site (patient ID)	Volume, cm^3^		OARs		
	GTV	CTV			
Head and neck			Parotid gland, Dmean, Gy	Larynx, Dmean, Gy	Spinal cord, Dmax, Gy
Cervical lymph node (P4)	NA	10.9	4.0	2.7	7.4
Cervical lymph node (P11)	2.3	216.9	17.8	15.7	14.4
Nasal cavity (P8)	128.3	331.9	13.2	9.7	16.7
Maxillary sinus (P5)	5.5	85.5	5.8	17.4	9.9
Masseter (P18)	29.6	88.5	30.4	6.5	14.2
Thorax			Lung, V20, %	Heart, Dmean, Gy	Spinal cord, Dmax, Gy
Axilla (P2)	19.8	118.9	0.7	0.2	4.2
Axilla (P6)	6.6	164.5	2.4	0.2	6.4
Axilla (P20)	NA	95.8	0.2	0.3	0.5
Axilla (P20)	NA	93.6	0.1	0.1	0.5
Mediastinum (P3)	24.6	283.3	2.2	9.5	14.7
Mediastinum (P4)	NA	65.1	0.2	0.3	16.8
Mediastinum (P12)	18.6	157.2	14.2	5.2	17.3
Mediastinum (P12)	80.1	258.1	6.6	14.4	21.3
Mediastinum (P15)	151.3	253.9	7.6	5.3	17.3
Mediastinum (P19)	13.5	343.7	27.8	16.6	19.6
Mediastinum (P20)	172.3	463.9	22.5	17.0	33.4
Mediastinum (P21)	2.8	51.4	2.9	0.3	15.1
Mediastinum (P22)	62.8	337.1	13.0	8.7	18.5
Arm (P4)	2.7	119.9	NA	1.1	2.5
Breast (P17)	191.5	781.4	6.9	1.1	18.7
Abdomen			Kidney, V20, %	Small intestine, Dmax, Gy	Spinal cord, Dmax, Gy
Spleen (P4)	12	172.4	4.3	6.5	9.8
Psoas major (P4)	26.7	137.4	NA	35.8	7.7
Buttock (P4)	9.6	233.3	NA	24.8	7.5
Back (P9)	191.9	692.6	23.3	30.9	24.0
Mesentery (P10)	86.7	258.8	2.2	38.9	15.1
Stomach (P16)	20.6	287.5	8.5	27.1	18.4
Retroperitoneum (P7)	95.7	685.6	18.6	37.7	19.5
Retroperitoneum (P13)	38.4	461.0	7.5	39.6	23.1
Retroperitoneum (P18)	116.3	225.0	NA	22.0	1.5
Retroperitoneum (P18)	8.0	155.8	6.2	39.2	15.9
Pelvic			Bladder, Dmean, Gy/V30, %	Head of femur, Dmax, Gy	Rectum, Dmean, Gy/V30, %
Prostate and bladder (P1)	134.7	512.6	29.1/24.1	19.3	23.6/23.6
Rectum and prostate (P14)	55.1	NA	16.2/5.2	13.8	23.5/32.6
Testicle (P7)	NA	145.1	5.9/NA	21.0	4.0/NA
Uterus (P11)	12.6	NA	5.1/1.9	8.7	3.30/NA
Groin lymph node (P8)	19.5	99.9	0.4/NA	1.5	0.7/NA
Groin lymph node (P18)	6.5	54.0	0.8/NA	20.0	1.8/NA

*Abbreviations*: *RT* radiotherapy, *Dmean* mean dose, *Dmax* maximal dose, *V20* percentage volumes receiving 20 Gy, *V30* percentage volumes receiving 30 Gy

## Discussion

Although the standard treatment for R/R aggressive B-cell lymphoma with late relapse (> 12 months) is dose-intensity chemotherapy followed by ASCT, most older patients are not considered ideal transplant candidates. The addition of consolidation or salvage RT unequivocally reduces the risk of local failure; however, a critical concern has been how to deliver RT in a short period of time, which did not delay effective systemic therapy. To our knowledge, this is the first study to provide valuable data of comprehensive hypofractionated RT for R/R aggressive B-cell lymphoma. Hypofractionated short-course RT exhibits excellent local control with mild toxicities.

The treatment options for R/R aggressive B-cell lymphoma show physician discrepancy and geographic variations between different countries or institutions, including chemotherapy alone, CAR T-cell therapy, and a sequential combination of chemotherapy and RT with or without ASCT [46–51]. Owing to heterogeneous treatments, a small number of patients receiving RT with different doses and fractions [28, 45, 52–54]. Recent studies have demonstrated that short-course bridging RT prior to CAR T-cell therapy provides excellent local control and a sustainable response. Theoretically, patients who will never be suitable for CAR T-cell therapy because of medical insurance-related issues and physical performance that may benefit from comprehensive hypofractionated RT [19–24, 55]. In this study, we present a homogenous cohort of 30 patients suffering from R/R aggressive B lymphoma. The comprehensive hypofractionated RT had an excellent response, with ORR and CR rates of 90% and 80%, respectively.

Salvage RT as part of potential treatment strategy is generally considered after second- or third-line systemic therapy. According to the ILROG guidelines for nodal NHL, patients with R/R disease unsuitable for transplantation may benefit from RT with doses up to 55 Gy [54]. Consequently, subsequent systemic treatment may be delayed for up to 6 weeks. The 2020 ILROG emergency RT guideline recommend hypofractionated schemes (36–39 Gy in 12–13 fractions or 30 Gy in six fractions) for chemorefractory NHL [44]. Recently, a cross-sectional study conducted by Memorial Sloan Kettering Cancer Center identified that the increased usage of hypofractionated RT was unique to sites affiliated with the hospital [54]. In our institution, the majority of lymphoma patients received IMRT or VMAT, and all R/R aggressive B-cell lymphoma received hypofractionated schemes since 2021 (36 Gy in 12 fractions). The median RT fraction was 12 in this study, fewer than the recent large retrospective study from British Columbia Cancer Agency (30–40 Gy in 15–20 fractions) [39].

As a non-cross-resistant therapy, RT could be a bridge to ASCT or CAR T-cell therapy to deepen remissions and improve cure rates. Metabolic tumor volume (MTV), as a representative of the total burden of disease, is the most important predictor of outcome in DLBCL and other lymphoma subtypes, regardless of the measurement method and study time points [56–59]. Here, we also showed that patients achieving CR after RT showed higher survival rates than those without CR. However, this high ORR rate was not entirely translated into an OS benefit. Out-of-field relapses continue to be a challenge, particularly in patients with advanced-stage disease, non-response to initial chemotherapy, or with multiple residual lesions at the time of RT. Similarly, 80% relapsed diseases occurred in new sites in our study. Therefore, the new agent should be added to RT to enhance the effects without obvious toxicity. At present, there are a number of clinical trials establishing the effects of immune checkpoint inhibitors in Hodgkin’s lymphoma [60–62]. However, DLBCL patients had a low response rate to the immune checkpoint inhibitor because chromosome 9p24.1 genetic alterations and PD-L1 or PD-L2 expression are rare in DLBCL. Hypofractionated RT can enhance the release of tumor antigens, increase tumor-reactive T cells, and work synergistically with immune checkpoint inhibitors in many solid tumors [63]. Presently, the combination of pembrolizumab and hypofractionated RT (20 Gy in five fractions) is in the phase 2 trial with R/R NHL (NCT04827862). To validate the above assumptions, we also performed a multicenter, single-arm, phase 2 study (ChiCTR2200060059) to assess the potential impact of Zimberelimab plus hypofractionated RT in patients with primary refractory DLBCL. The study is currently enrolling patients. The clinical benefit of hypofractionation RT and immune checkpoint inhibitors needs to be further investigated in these prospective studies.

This study has some limitations, mainly related to its retrospective nature. While the data support important findings regarding a high response rate and mild toxicities with hypofractionated RT, the treatments were not randomly assigned. Additionally, none of the patients received CAR T-cell therapy. Although CAR T-cell therapy has been recommended based on the guidelines, is not cost effective and may not be feasible for most patients in China. In fact, the data we observed that could provide an option for CAR T-cell therapy-eligible patients. Furthermore, because of the short follow-up period, we were unable to adequately assess the late toxicities. However, hypofractionated RT has been widely employed in several types of solid tumors with long-term follow-up. We believe that hypofractionated RT is efficacious and safe.

## Conclusion

We showed that hypofractionated RT achieved high response rates and was well tolerated in patients with R/R aggressive B-cell lymphoma. These findings provide additional evidence supporting hypofractionated RT as a treatment for reduction of tumor burden in aggressive B-cell lymphomas.

## Data Availability

The datasets used and/or analysed during the current study are available from the corresponding author on reasonable request.

## Abbreviations

NHL: Non-Hodgkin lymphoma
R-CHOP: Rituximab, cyclophosphamide, doxorubicin, vincristine, and prednisone
R/R: Refractory or relapsed
ASCT: Autologous stem-cell transplantation
CAR: Chimeric antigen receptor
RT: Radiotherapy
PET: Positron emission tomography
CNS: Central nervous system
CR: Complete response
EPOCH-R: Etoposide, prednisone, vincristine, cyclophosphamide, doxorubicin, rituximab
ILROG: International Lymphoma Radiation Oncology Group
ISRT: Involved-site radiation therapy
MRI: Magnetic resonance imaging
GTV: Gross tumor volume
CTV: Clinical target volume
PTV: Planning target volume
OAR: Organs at risk
PR: Partial response
PFS: Progression-free survival
OS: Overall survival
DLBCL: Diffuse large B-cell lymphoma
PMBL: Primary mediastinal large B-cell lymphoma
PD: Progressive disease
IMRT: Intensity-modulated radiation therapy
VMAT: Volumetric-modulated arc therapy
ORR: Objective response rate
Dmean: Median mean dose
Dmax: Median maximal dose
V20: Volume irradiated by 20 Gy or more
